# Triptolide suppresses IL-1β-induced expression of interleukin-8 by inhibiting ROS-Mediated ERK, AP-1, and NF-κB molecules in human gastric cancer AGS cells

**DOI:** 10.3389/fonc.2024.1498213

**Published:** 2025-01-30

**Authors:** Shinan Li, Dhiraj Kumar Sah, Archana Arjunan, Mohamed Yazeer Ameer, Bora Lee, Young-Do Jung

**Affiliations:** ^1^ Department of Biochemistry and Molecular Biology, School of Basic Medicine, Shanxi Medical University, Taiyuan, China; ^2^ Department of Biochemistry, Chonnam National University Medical School, Hwasun, Republic of Korea

**Keywords:** angiogenesis, ERK1/2, gastric cancer, Interleukin-1ß, reactive oxygen species, triptolide, Interleukin-8

## Abstract

Triptolide, the major component of Chinese herbal medicine Tripterygium wilfordii Hook F, possesses potent anticancer and anti-inflammatory effects. IL-8, a proinflammatory cytokine, is associated with cancer cell proliferation and angiogenesis. Here, we found that Triptolide has an inhibitory effect on IL-1β-induced IL-8 expression in human gastric cancer cells, via the suppression of reactive oxygen species (ROS) production, AP-1, and NF-κB activation, which in turn affects human endothelial cell angiogenetic activity in tumor microenvironments. Human gastric AGS cells were treated with IL-1β (10 ng/mL) and Triptolide (0–20 nM), and the ROS generation, ERK, AP-1, and NF-κB signaling were all investigated. These results demonstrate that Triptolide inhibits the IL-1β-induced IL-8 expression in gastric cancer cells by inhibiting ROS production and angiogenesis, via the dose-dependent attenuation of ERK, AP-1, and NF-κB activation. In this study, we showed that Triptolid inhibits ROS/ERK-mediated AP-1 and ROS-mediated NF-κB axes potentially leading to an improved treatment outcome for gastric cancer and its associated tumor microenvironment.

## 1 Introduction

Gastric cancer is a global health issue and is ranked the fifth most common cancer and the third most lethal worldwide (1). Gastric cancer (GC), also known as gastroesophageal adenocarcinoma or stomach cancer, results from cancerous growth in the digestive tract (2). It is a geographically diverse disease (3), with the highest incidence rate in Asia/Pacific regions, where one million cases are diagnosed each year (4), and it has a global mortality rate of 783,000 deaths (5, 6). Therefore, it is essential to understand the mechanisms that cause and develop gastric cancer in order to refine its prevention and treatment. There is growing evidence that chemokines are linked to cancer and could be used as a potential biomarker for early diagnosis and prognosis predictions (7). Recent research has confirmed that interleukin-1β (IL-1β) is associated with the facilitation of gastric cancer following *Helicobacter pylori* (HP) infection, and chronic inflammation plays a critical role in the growth and proliferation of GC. IL-1β induces gastric cancer by blocking the production of gastric acid, causing epigenetic changes, stimulating angiogenesis, attracting adhesive factors, and releasing other inflammatory factors, such as interleukin-8 (IL-8/CXCL8) (8–11). Although IL-1β increases IL-8 expression in various cells, including endothelial, epithelial, and smooth muscle cells, the molecular mechanism underlying IL-1β-induced IL-8 expression in gastric cancer is unknown.

A growing body of evidence suggests that IL-8/CXCL8 has chemotactic, proangiogenic, and tumorigenic properties (12, 13). The primary regulators of IL-8 expression are transcription factors including nuclear factor-κB-mediated transcription factors (NF-κB) (14). When activated through CXCR1 and CXCR2 (cell-surface G protein-coupled receptors), IL-8 activates phosphatidylinositol-3-kinase or phospholipase C, which activates the Akt/PKB, mitogen-activated protein kinase (MAPK), and protein kinase C (PKC) signaling pathways, which are mainly responsible for inflammation (15, 16). Evidence indicates that IL-8 derived from tumors significantly impacts the tumor microenvironment (17–19). Its role is critical in maintaining cancer cells in the epithelial–mesenchymal transition (EMT) trait (20), which confers proliferation and invasiveness, as well as promoting angiogenesis in various cancers (21, 22). However, there is only limited research available on the relationship between IL-1ß and IL-8, as well as the molecular mechanisms involved in gastric cancer progression. Therefore, it has been suggested that unraveling and targeting CXC-chemokine signaling may have significant implications in halting disease progression and assisting in sensitizing tumors to chemotherapeutic and biological agents, given the multiple effects of IL-8 signaling on gastric cells within the tumor microenvironment.

Nonetheless, therapeutic interventions for advanced/metastatic gastric cancer have evolved dramatically in recent years. Indeed novel drugs have emerged at an exponential rate, primarily focusing on inhibiting the oxidative stress-induced inflammatory response, which counteracts aberrant cancer signaling by inhibiting cancer growth and proliferation. This has provided oncologists with a wide range of advanced treatment options against late-stage GC. Triptolide is a primary bioactive compound derived from Tripterygium wilfordii Hook F, which belongs to the Celastraceae family and the Tripterygium genus, and has been found in Southern China. Its roots have been used in a myriad of practices to “relieve stasis and internal warmth,” among many other diseases diagnosed by Traditional Chinese medicine practitioners. Recent studies have shown that Triptolide inhibits tumor proliferation and metastasis, induces apoptosis, and enhances the impact of other therapeutic interventions in various types of cancers (23–26). Thus, considering all this, the objective of this study was to determine the effect of Triptolide on IL-1β-induced IL-8 expression in gastric cancer by modulating ROS-mediated AP-1/NF-κB and ERK signaling.

## 2 Materials and methods

### 2.1 Cell culture and culture conditions

Human gastric cancer AGS cell line and endothelial cell line EA.hy926 were obtained from the American Type Culture Collection (Manassas, VA, USA). The cells used in this study were cultured under the same conditions described in our previous study (27). Briefly, the effect of Triptolide on the IL-1β-stimulated expression of IL-8 was examined by harvesting the cells at different intervals and measuring the level of IL-8 mRNA by RT-PCR. To determine the effects of Triptolide in IL-1β (R&D Systems, Inc., Minneapolis, MN, USA) induced ERK1/2 activation, cells were harvested at various intervals, and the phosphorylated and total protein levels were determined by Western blot.

### 2.2 Western blot analysis

Protein extraction and Western blot analysis were performed as previously described (27). Several primary antibodies were used (Cell Signaling Technology, Danvers, MA, USA): anti-phospho-ERK1/2, anti-phospho-p65, anti-phospho-c-Fos, and anti-phospho-c-Jun. The secondary antibody was horseradish peroxidase-labeled anti-rabbit immunoglobulins from donkey (Amersham Corp., Arlington Heights, IL, USA), which was used at a dilution of 1:3000. Protein bands were visualized using a Western chemiluminescent HRPV substrate (Millipore Corporation, Billerica, MA, USA). The total protein levels were assayed by washing the blotted membrane with stripping solution [100 mM 2-mercaptoethanol, 2% sodium dodecyl sulfate, and 62.5 mM Tris–HCl (pH 6.7)] for 30 min at 50°C, and then, the membrane was applied with anti-ERK1/2 and anti-p65 antibodies (Cell Signaling Technology, Danvers, MA, USA) diluted to 1:2000.

### 2.3 Measurement of intracellular H_2_O_2_

Intracellular H_2_O_2_ was measured using 5-(and 6)-carboxyl-2’,7’-dichlorodihydrofluorescein diacetate (DCFDA, Grand Island, NY, USA), according to the same procedure in a previous study (28). To investigate the role of Triptolide in IL-1β-induced ROS production, cells were grown in serum-starved DMEM medium supplemented with 0.5% FBS for an additional 2 days. Then, the cells were stabilized in serum-free DMEM medium without phenol red for at least 30 min before exposure to IL-1β for 0–20 min. To assess the effect of Triptolide, the cells were pretreated with Triptolide for 30 min. Then, the cells were incubated with the H_2_O_2_-sensitive fluorophore DCFDA (10 μM) for 30 min and immediately observed under a laser-scanning confocal microscope. DCF fluorescence was excited at 458 nm using an argon laser, and the evoked emission was filtered with a 538 nm long pass filter.

### 2.4 Measurement of IL-8 promoter assay

The transcriptional regulation of IL-8 by IL-1β was examined following the transient transfection of an IL-8 promoter-luciferase reporter construct (pGL2-IL-8) (29). AGS cells (5 × 10^5^) were seeded and grown to 60–70% confluence, and then, pRLTK (an internal control plasmid containing the herpes simplex thymidine kinase promoter linked to the constitutively active Renilla luciferase reporter gene) and pGL2-IL-8 were cotransfected into the cells using Lipofectamine (Invitrogen, Carlsbad, CA, USA). According to the manufacturer’s protocol, pRLTK and pGL2 were cotransfected as the negative control. Cells were incubated in the transfection medium for 20 h and pretreated with different doses of Triptolide for 1h prior to their incubation with IL-8 for 4 h. After incubation, the cells were harvested and lysed with passive lysis buffer (Dual-Luciferase Reporter Assay System; Promega, Madison, WI, USA), and luciferase activity was measured using a luminometer.

### 2.5 Determination of the effect of AGS-derived conditioned medium on the proliferation of EA.hy926 cells

Conditioned medium (CM) derived from AGS cells was prepared as follows: Cells were grown to 95–100% confluence and incubated for 24 h in DMEM medium with 1% FBS and 10 ng/mL IL-1β or Triptolide pretreated for 1h. After incubation, supernatants [CM or CM(T)] were collected, centrifuged, and stored at -20°C. To determine the effect of CM on endothelial cell proliferation, EA.hy926 cells (5 × 10^3^) were plated in 96-well plates (Falcon Laboratories, McLean, VA, USA) and incubated for 24 h with DMEM containing 10% FBS. The medium was replaced with CM, and the cells were incubated for a further 24 h. The neutralizing effect of the anti-IL-8 antibody on the proliferation activity of CM was determined by incubating the cells with CM and CM(T) after CM and CM(T) were treated for 1h with 1 μg/mL neutralizing antibody to IL-8 (R&D Systems, Minneapolis, MN, USA) Cell proliferation was determined using the MTT assay in which the MTT was converted to formazan granules in the presence of molecular oxygen. After incubation, 50 μL of 5 mg/mL MTT was added to each well of the 96-well plates and incubated at 37°C for 2 h. The formazan granules obtained were dissolved in 100% DMSO, and the absorbance was detected at 562 nm using a 96-well ELISA reader (Biotek Inc., Winooski, VT, USA).

### 2.6 Angiogenesis assay

Conditioned medium (CM) derived from AGS cells was prepared as previously described (30). Briefly, AGS cells were grown to 95–100% confluency and incubated for 24 h in DMEM medium with 1% FBS and 10 ng/mL IL-1β or Triptolide pretreated for 1h. After incubation, the supernatants were collected, centrifuged, and stored at -80°C until use. Corning^®^ Matrigel^®^ Basement Membrane Matrix (9.1 mg/mL; Sigma-Aldrich, St. Louis, MO, USA) was loaded in a 96-well plate (60 μL/well) and incubated at 37°C for at least 30 min. EA.hy926 cells were plated (5 × 10^3^) on the prepared thin Matrigel 96-well plate with 50 μL DMEM 10% FBS media for 4 h before the EA.hy926 cells were incubated for 6 h with the prepared CM. The IL-8 anti-body (1 μg/mL; R&D Systems, Minneapolis, MN, USA) and a non-specific IgG (R&D Systems, Minneapolis, MN, USA) were used. The synthesized IL-8 (1 ng/mL; Santa Cruz Biotechnology, Santa Cruz, CA, USA) along with the control CM was added to the EA.hy926 cells as the positive control to evaluate the IL-8 effect on the endothelial cell angiogenic activity. Quantification of the nodes, junctions, branches, and segments was conducted using an Angiogenesis Analyzer (software ImageJ; http://image.bio.methods.free.fr/ImageJ/?Angiogenesis-Analyzer-for-ImageJ&lang=en&artpage=3-6#outil_sommaire_3, accessed on 20, February 2023).

### 2.7 Statistical analysis

Each value represents three individual experiments and is presented as the mean ± standard deviation (SD). The results were analyzed using Graph Pad Prism software (Version 8.0). The differences between the two datasets were analyzed using a t-test. The statistically significant differences described in the text correspond to a p-value of < 0.05.

## 3 Results

### 3.1 Triptolide inhibits IL-1β-induced IL-8 expression in gastric cancer AGS cells

**Figure 1:**
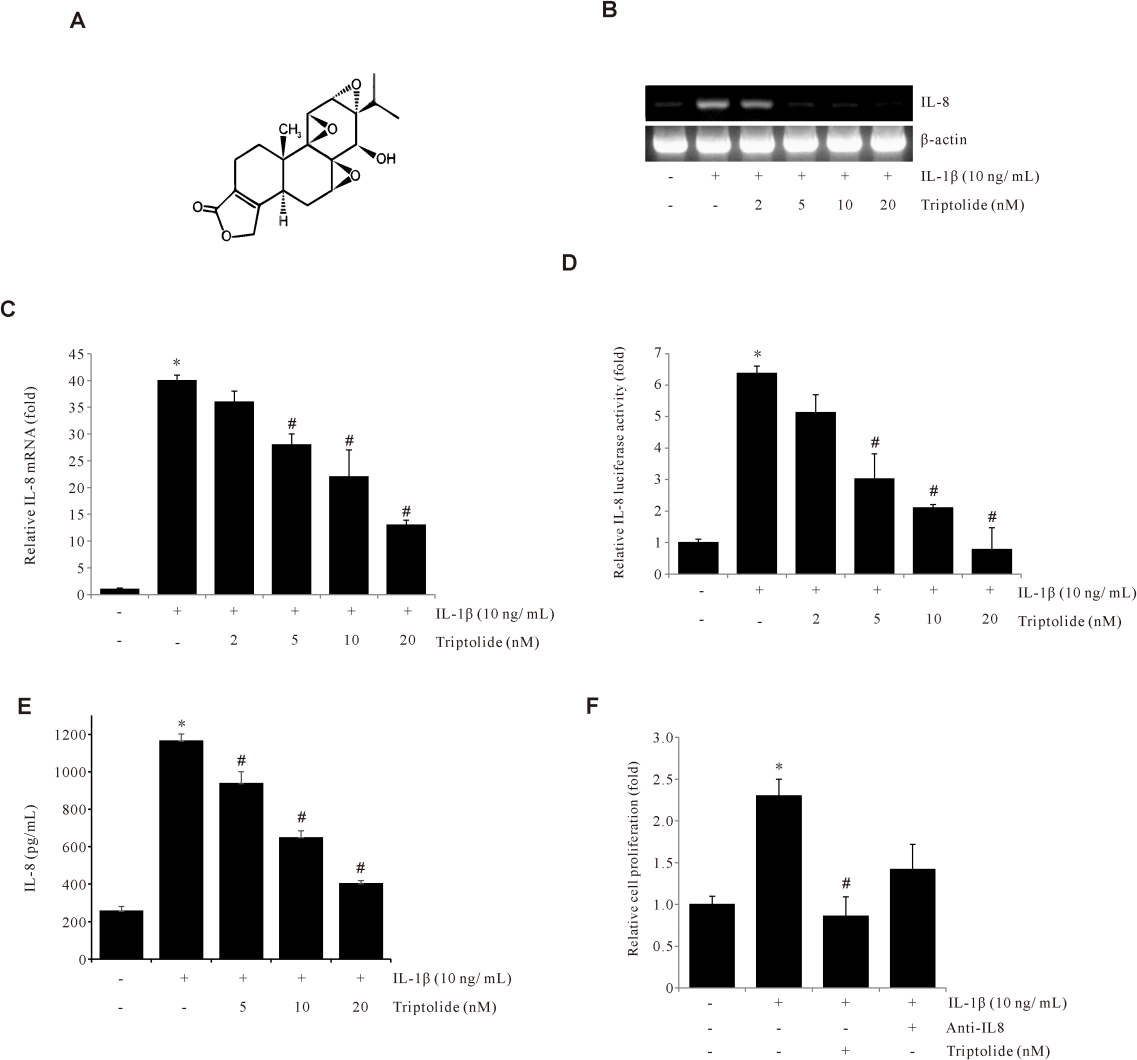
Triptolide inhibits IL-1β-induced IL-8 expression in AGS cells. **(A)** The chemical structure of Triptolide. **(B, C)** AGS cells were pretreated with Triptolide (2, 5, 10, and 20 μM) for 1 h, followed by treatment with 10 ng/mL IL-1β for 4 h, and IL-8 mRNA levels were determined by RT-PCR and qPCR. **(D)** AGS cells were transiently transfected with the pGL2-IL-8 promoter-luciferase construct. The transfected cells were pretreated with Triptolide (2, 5, 10, and 20 nM) for 1 h, followed by treatment with 10 ng/mL IL-1β for 4 h, and the luciferase activity was measured using a luminometer. **(E)** AGS cells were pretreated with Triptolide (5, 10, and 20 nM) and incubated with 10 ng/mL IL-1β for 24 h, followed by ELISA to determine the amount of IL-8 secretion. **(F)** AGS cells were pretreated with Triptolide or IL-8 antibody and incubated with 10 ng/mL IL-1β for 24 h, and the cell viability was determined by MTT assay. The data are presented as the mean ± standard deviation (SD) from triplicate measurements. *p < 0.05 versus control; ^#^p < 0.05 versus treatment with IL-1β only.

To determine the effect of Triptolide on IL-1β-induced IL-8 expression in human gastric cancer AGS cells, the cells were pretreated with Triptolide (**Figure 1A**), and the levels of IL-8 mRNA were determined by RT-PCR and qPCR analyses. The RT-PCR and qPCR results showed that Triptolide decreased IL-1β-induced IL-8 mRNA expression in a dose-dependent manner (**Figures 1B, C**). Additionally, Triptolide also decreased IL-8 promoter activity in a dose-dependent manner (**Figure 1D**). Similar results were observed in the ELISA evaluation (**Figure 1E**). Additionally, Triptolide inhibited IL-1β-induced AGS cell proliferation (**Figure 1F**). Collectively, these results demonstrated that Triptolide suppresses the IL-1β-induced IL-8 expression in gastric cancer AGS cells and further inhibited the IL-1β-induced cancer cell proliferation.

### 3.2 Triptolide suppresses IL-1β-induced IL-8 expression by inhibiting ROS generation

**Figure 2:**
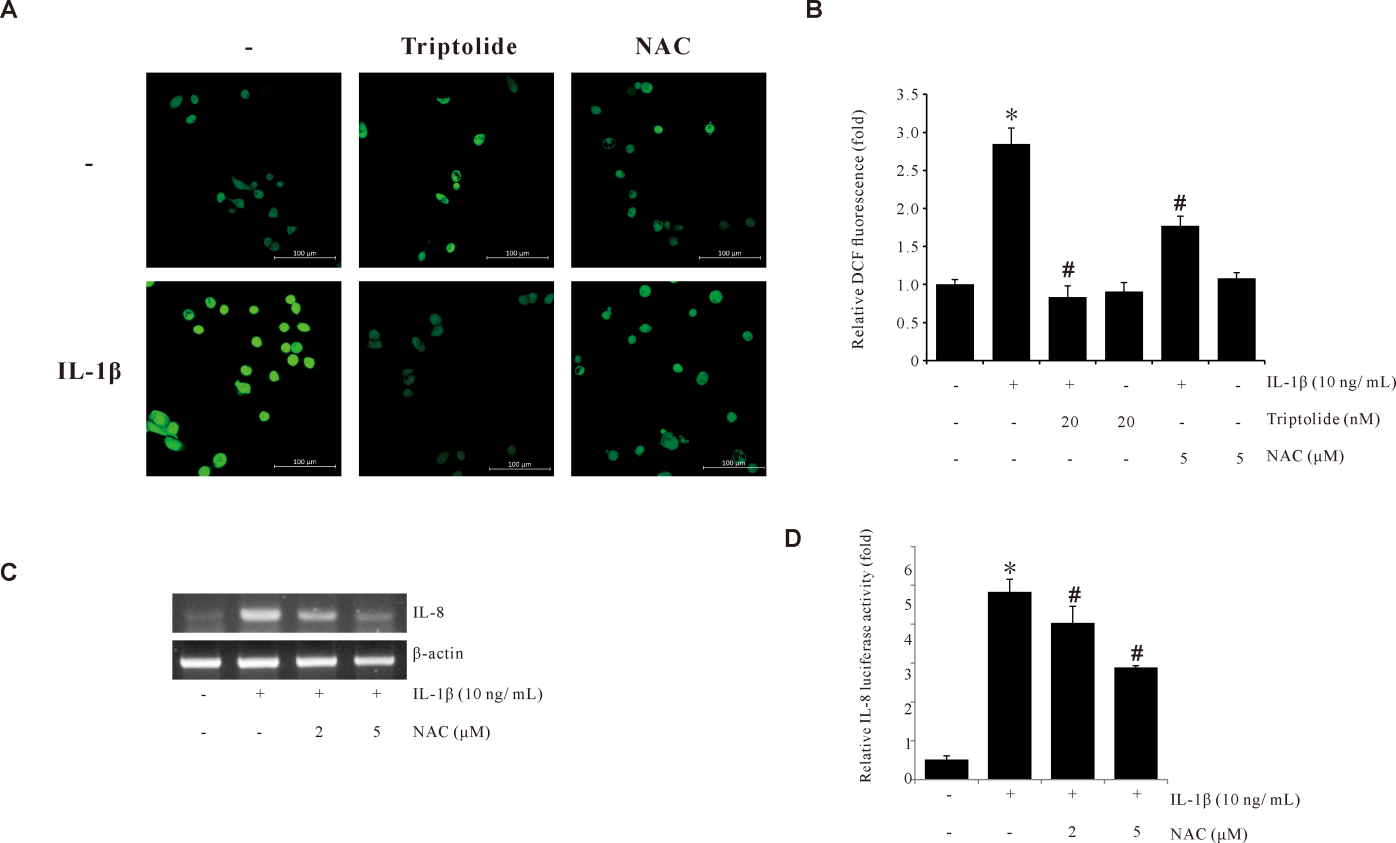
Triptolide inhibits IL-1β-induced ROS production in AGS cells. **(A)** Synchronized quiescent AGS cells pretreated with 20 nM Triptolide and 5 mM NAC for 1 h prior to IL-1β treatment for 30 min. Then, the cells were incubated in the dark for 10 min with 10 µM H2DCFDA. The H2DCFDA fluorescence was imaged using a confocal laser scanning fluorescence microscope. **(B)** ROS level quantifications detected by DCFDA fluorescence intensities. **(C)** AGS cells pretreated with 5 mM NAC for 1 h were incubated with IL-1β for 4 h. After incubation, IL-8 mRNA levels were determined by RT-PCR. **(D)** AGS cells were transiently transfected with a pGL2-IL-8 promoter-luciferase construct. The transfected cells were pretreated with NAC (2 and 5 μM) for 1 h, followed by treatment with 10 ng/mL IL-1β for 4 h, and the luciferase activity was measured using a luminometer. The data are presented as the mean ± standard deviation (SD) from triplicate measurements. *p < 0.05 versus control; ^#^p < 0.05 versus treatment with IL-1β only.

To determine the effect of Triptolide on ROS production, the ROS production levels in AGS cells treated with IL-1β, in the presence or absence of Triptolide, were assessed using the H2DCFDA assay. As shown in **Figures 2A, B**, Triptolide suppressed the IL-1β-induced ROS production levels. We further observed that pretreatment of AGS cells with N-acetylcystein (NAC), a ROS scavenger, abrogated the IL-1β-induced IL-8 mRNA expression and the IL-1β-induced IL-8 promoter activity (**Figures 2C, D**). These results suggested that the suppression of IL-1β-induced IL-8 expression by Triptolide is mediated by inhibiting ROS production in human gastric cancer AGS cells.

### 3.3 Triptolide suppresses IL-1β-induced IL-8 expression by inhibiting the AP-1 transcription factor via the inhibition of c-Fos and c-Jun phosphorylation

**Figure 3:**
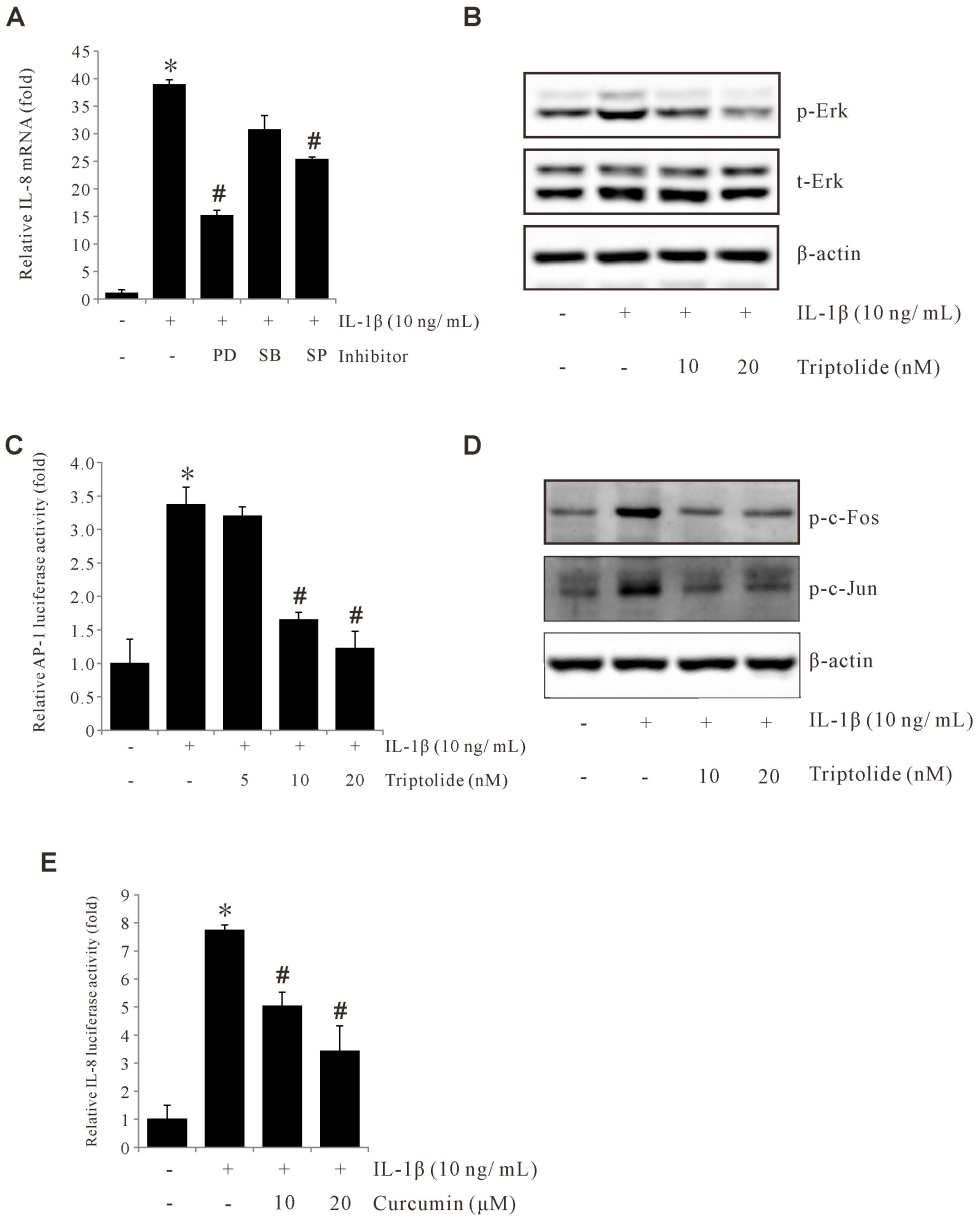
Triptolide inhibits IL-1β-induced IL-8 by suppressing ERK1/2, c-Fos, c-Jun, and AP-1 activation. **(A)** AGS cells were pretreated with 30 µM PD98059, 20 µM SB203580, and 30 μM SP600125 for 1 h and incubated with 10 ng/mL IL-1β for 4 h, and IL-8 mRNA levels were determined by qPCR. **(B)** AGS cells were pretreated with Triptolide (10 and 20 nM) followed by 10 ng/mL IL-1β treatment for 30 min, and the extracted proteins were analyzed using Western blotting. **(C)** AGS cells were transiently transfected with AP-1 promoter-luciferase construct. The transfected cells were pretreated with Triptolide (5, 10, and 20 nM) for 1h, followed by treatment with 10 ng/mL IL-1β for 4 h, and the luciferase activity was measured using a luminometer. **(D)** AGS cells were transiently transfected with a pGL2-IL-8 promoter-luciferase construct. The transfected cells were pretreated with curcumin (10 and 20 μM) for 1 h, followed by treatment with 10 ng/mL IL-1β for 4 h, and the luciferase activity was measured using a luminometer. **(E)** AGS cells were transiently transfected with pGL2-IL-8 promoter luciferase construct. The transfected cells were pretreated with curcumin (10 and 20 μM) for 1h, followed by treatment with 10 ng/mL IL-1β for 4 h, and the luciferase activity was measured using a luminometer. The above data represent the mean ± standard deviation (SD) from triplicate measurements. *p < 0.05 versus control; ^#^p < 0.05 versus treatment with IL-1β only.

The molecules c-Fos and c-Jun are protein subunits of AP-1 and have been previously reported as playing an important role in gastric cancer development. Due to the crucial role that MAPK plays in AP-1 activation, we examined the effect of MAPK on IL-1β-induced IL-8 expression. We found that among the MAPK inhibitors we tested, only the ERK1/2 inhibitor (PD98059) was able to inhibit IL-1β-induced IL-8 expression in AGS cells (**Figure 3A**). Further, Western blotting was used to confirm that Triptolide blocked the IL-1β-induced ERK1/2 phosphorylation in AGS cells (**Figure 3B**). These results indicate that the ERK1/2 pathway is crucial for IL-1β-induced IL-8 expression, which can be abrogated by Triptolide. Next, we examined if Triptolide can suppress AP-1 activity. As shown in **Figures 3C, D**, Triptolide inhibited transcriptional activity of AP-1 and suppressed c-Fos and c-Jun phosphorylation as well. Also, AP-1 inhibitor curcumin significantly reduced L-1β-induced IL-8 promoter activity (**Figure 3E**). Overall, these results demonstrate that Triptolide can inhibit IL-1β-induced IL-8 expression by suppressing AP-1 activation.

### 3.4 Triptolide suppresses IL-1β-induced IL-8 expression by inhibiting the NF-κB transcription factor via the inhibition of p65 phosphorylation

**Figure 4:**
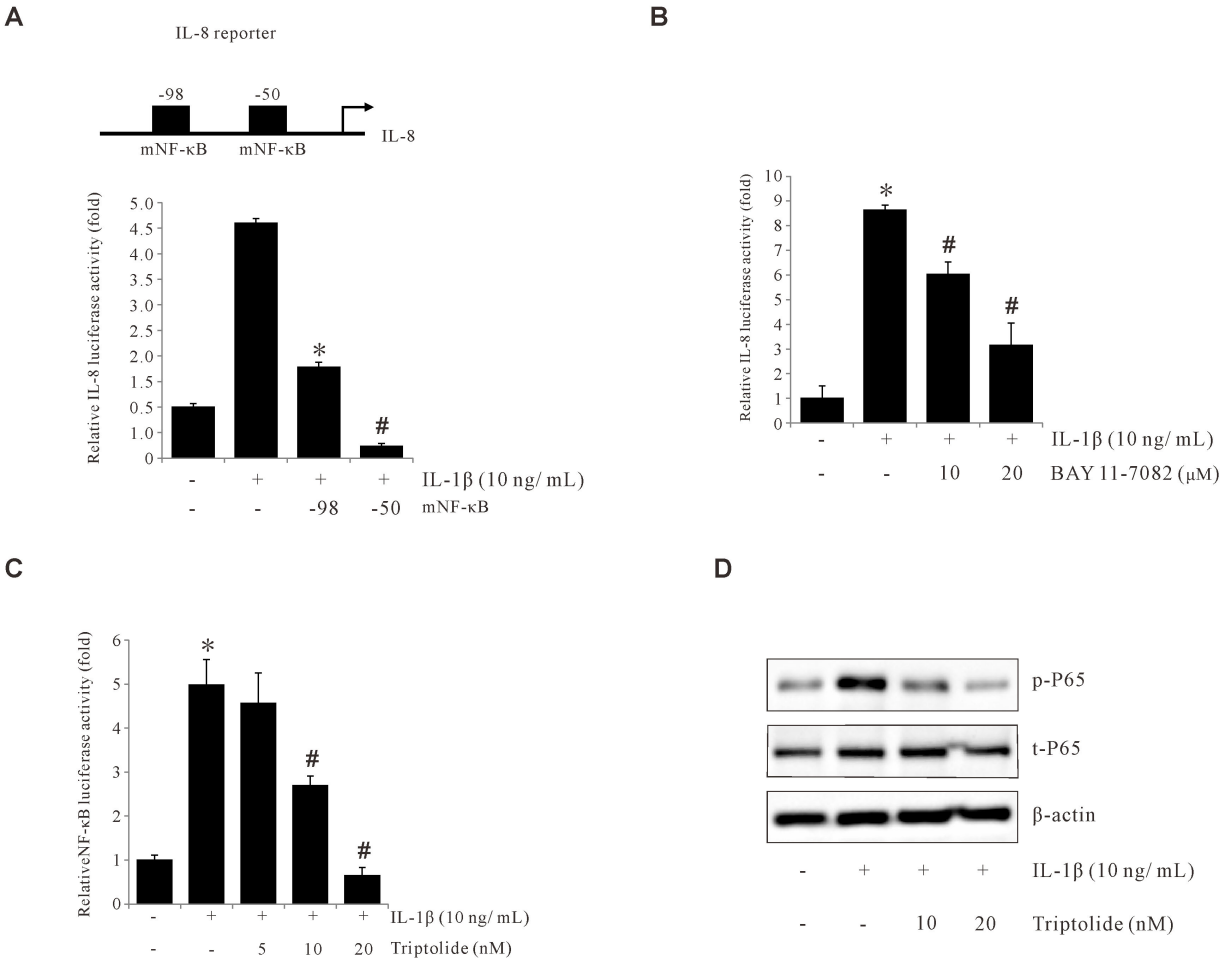
Triptolide inhibits IL-1β-induced IL-8 by suppressing p65 and NF-κB activation. **(A)** The IL-8 promoter was sequentially deleted in the 5′-flanking region, and the promoter-luciferase construct was transiently transfected into AGS cells. The transfected cells were incubated with 10 ng/mL IL-1β, and the luciferase activity was measured using a luminometer. Data represent the mean ± standard deviation (SD) from three experimental trials. *p < 0.05 versus IL-1β only; # p < 0.05 versus –98. **(B)** AGS cells were transiently transfected with a pGL2-IL-8 promoter-luciferase construct. The transfected cells were pretreated with BAY11-7082 (10 and 20 μM) for 1 h, followed by treatment with 10 ng/mL IL-1β for 4 h, and the luciferase activity was measured using a luminometer. **(C)** AGS cells were transiently transfected with an AP-1 promoter-luciferase construct. The transfected cells were pretreated with Triptolide (5, 10, and 20 nM) for 1 h, followed by treatment with 10 ng/mL IL-1β for 4 h, and the luciferase activity was measured using a luminometer. **(D)** AGS cells were pretreated with Triptolide (10 and 20 nM) followed by 10 ng/mL IL-1β treatment for 30 min, and extracted proteins were analyzed using Western blotting. The data are presented as the mean ± standard deviation (SD) from triplicate measurements. *p < 0.05 versus control; ^#^p < 0.05 versus treatment with IL-1β only.

To find out the important DNA element for the IL-1β-induced activation of IL-8 promoter, IL-8 promoter activities were analyzed with deletion assays. Notably, a remarkable reduction was observed following the deletion of the upstream region at nucleotide positions –98 bp and –50 bp, after IL-1β treatment (**Figure 4A**), indicating that the –98 to –50 position is critical for the IL-1β-induced IL-8 promoter activity. This critical region for IL-1β-induced activity lies within a known NF-κB binding element on IL-8 promoter, spanning from -80 to -70. To examine if this reduced promoter activity is through NF-κB binding, BAY (AY11-7082, an NF-κB inhibitor) was used with the IL-8 promoter assay. As shown in **Figure 4B**, BAY significantly suppressed IL-1β-induced IL-8 promoter activity. Consistently, Triptolide inhibited IL-1β-induced NF-κB activation and p65 phosphorylation (**Figures 4C, D**). Collectively, these data suggest that IL-1β induces IL-8 expression by activating NF-κB and that Triptolide inhibited IL-1β-induced IL-8 expression by suppressing NF-κB activation.

### 3.5 Triptolide suppresses the IL-1β-induced angiogenesis activity in gastric cancer AGS cells

**Figure 5:**
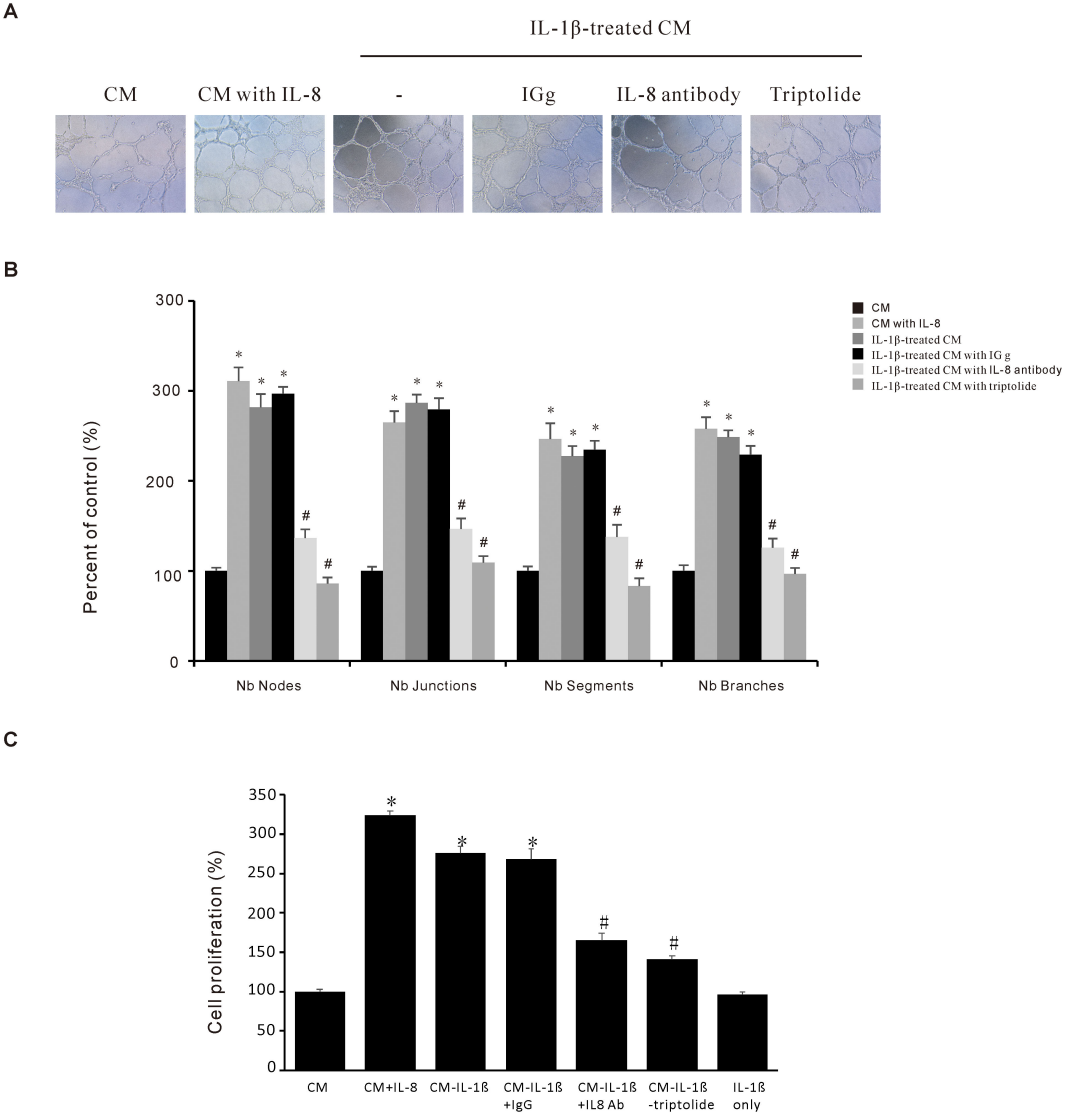
Triptolide suppresses the IL-1β-induced angiogenesis activity in gastric cancer AGS cells. **(A)** Representative images (10 ×) of endothelial EA. hy926 cells tube formations. The EA. hy926 cells were grown in a Matrigel-coated plate for 24 h and incubated with CM obtained from Triptolide-pretreated and IL-1β-stimulated AGS cells. After 6 h, the cells were observed and counted using a Nokia microscope **(B)** Quantitative data of the EA. hy926 tube formation. **(C)** EA.hy926 cells were incubated with 1000 pg/mL IL-8, 10 ng/mL IL-1β, or conditioned medium (CM) for 24 h, in the presence or absence of 1 mg/mL anti-IL-8 antibody, then cell proliferation was assessed. The data are presented as the mean ± standard deviation (SD) from triplicate measurements. *p < 0.05 versus control; ^#^p < 0.05 versus treatment with IL-8 only.

Angiogenesis is a vital process in the tumor microenvironment that plays a pivotal role in cancer progression. In the current study, we used the endothelial EA.hy926 cell line to determine the effect of Triptolide on IL-1β-induced angiogenesis *in vitro*. As shown in **Figures 5A, B**, the angiogenic activity assessed by tube formation was significantly promoted after treatment with IL-8, or IL-1β-treated conditioned medium (CM) compared to control-CM. Furthermore, this IL-1β-induced angiogenic activity of EA.hy926 cells is decreased after either IL-8 antibody or Triptolide treatment. And we utilized EA.hy926 endothelial cells to investigate whether modulation of IL-8 expression in AGS cells could influence endothelial cell proliferation. Our result revealed that conditioned medium (CM) from IL-1β-treated AGS cells significantly enhanced EA.hy926 cell proliferation, an effect that was effectively suppressed by triptolide (**Figure 5C**). These findings suggest that Triptolide could inhibit IL-1β-induced endothelial angiogenesis by suppressing IL-8 expression in gastric cancer AGS cells, thus having a potential impact on the tumor microenvironment.

## 4 Discussion

Herbal compounds, particularly those based on traditional medicines, have demonstrated their potential to serve as effective treatments. The pharmacologically active ingredients in herbal medicines have been extensively studied, leading to their widespread use in contemporary medical therapies. Research has shown that these herbal compounds can play crucial role in improving health outcomes and providing relief from a variety of ailments (31). Triptolide (diterpene triepoxide) (**Figure 1A**) is derived from Tripterygium wilfordii Hook F, a traditional Chinese perennial herb whose extracts have been used for centuries in antioxidative (32), anti-inflammatory (33), anticancer (34, 35), and immunosuppressive therapies (36, 37). Triptolide and its analogs exhibit potent bioactivities against various cancers, including breast (38, 39), lung (40–42), gastric (43, 44), and colon cancers (45–47).

The chemokine IL-8 is widely acknowledged to play an essential role in tumor sustenance, invasion, and angiogenesis, as well as immune suppression via various signaling pathways. Therefore, targeting IL-8 is the ideal therapeutic approach to prevent cancer progression. In this study, we found that IL-1β increased IL-8 expression, while Triptolide reduced this IL-1β-induced IL-8 expression in AGS gastric cancer cells (**Figure 1**). We further investigated the molecular mechanism of anticancer effect of Triptolide on gastric cancer.

The main etiological factor for gastric cancer is chronic Helicobactor pylori (HP) infection. IL-1β, a pluripotent proinflammatory cytokine, plays a crucial role in the pathogenesis of HP-induced mucosal inflammation and gastric carcinogenesis (48, 49). Our findings demonstrated that IL-1β increased IL-8 expression by upregulating reactive oxygen species (ROS), ERK, AP-1, and NF-κB signaling pathways in gastric cancer cells, and that all these signaling pathways can be inhibited by Triptolide treatment.

A variety of intracellular signals have been proposed to mitigate the impacts of IL-1β, including the activation of MAPK, the secretion of arachidonic acid, the hydrolysis of sphingomyelin, and the formation of ROS (49, 50). The IL-1β signal is initiated by the binding of IL-1β to their receptor (IL-1R1) and coreceptors (IL-1RAP), resulting in the formation of a trimeric complex and activation of TRAF6 signaling. TRAF6 signaling can occur via two main pathways: IKK–IB–NF-κB and/or MKK–MAPK/JNK/ERK. Phosphorylated TAK1 activates the inhibitor of nuclear factor kappa-B kinase subunit beta (IKKβ) and activated IKK phosphorylates the nuclear factor kappa-B inhibitor (IkB), which is degraded, allowing NF-κB to be released and migrate to the nucleus. TAK1 also activates MAPK p38, JNK, and ERK (51, 52). The IL-8 gene promoter contains the binding sites for AP-1, NF-κB, and CERB (49, 53). Our results also confirmed that IL-1β induced NF-κB activation to promote IL-8 promoter activity (**Figures 4A, B**), while Triptolide inhibited NF-κB activation. Alternatively, several studies have suggested that ROS can activate the c-Jun NH2-terminal kinases (54) and NF-κB pathways (55). Our findings also demonstrated that IL-1β induced ROS production in AGS cells, whereas Triptolide treatment suppressed IL-1β-induced ROS generation (**Figure 2**).

Haifeng Zhang et al. mentioned that Triptolide inhibited the IL-1β expression in an ulcerative colitis mouse model (56). Their findings suggest that Triptolide might inhibit IL-1β expression directly, which in turn inhibits IL-8 expression in AGS gastric cancer cells. Our previous study demonstrated that Triptolide inhibits uPAR-induced ERK, NF-κB, AP-1, and ROS production in AGS cell lines (34). Furthermore, another study supported these findings, whereby Triptolide pretreatment inhibited ROS production by suppressing the Nrf2 and NF-κB transactivation in a caerulein-induced acute pancreatitis animal model and an *in vitro* cell model (57). All these reports support our findings that Triptolide reduces IL-1β-induced IL-8 expression by inhibiting ROS production, and activation of ERK1/2, AP-1 and NF-κB.

Triptolide inhibits the NF-κB signaling in various ways. NF-κB is a heterogeneous dimer, consisting of p50 and p65, which promotes cell proliferation, prevents apoptosis, and is involved in tumor development (58). Triptolide inhibits NF-κB via caspase activation by blocking the transactivation impact of the NF-κB p65 subunit (59). Triptolide also influences NF-κB indirectly via the AKT/GSK3/mTOR pathway and downstream of the PI3K/Akt signaling pathway (60). TPL also inhibits complex I in the mitochondrial respiratory chain (MRC), which inhibits the production of ROS as well as the activation of NF-κB (61). Tao et al. reported that Triptolide decreased the IL-1β-induced NF-κB DNA binding capacity and cytosolic amount of p-IκBα in subepithelial myofibroblasts (62). Furthermore, Triptolide inhibited matrix metalloproteinase-9 expression and invasion by inhibiting NF-κB and AP-1 expression in breast cancer cells (63). Similarly, Weiwei Yuan et al. reported that pretreatment with Triptolide increased apoptosis in TNF-α-induced gastric cancer cells by disrupting the H19/miR-204-5p/NF-κB/FLIP axis (64).

**Figure 6:**
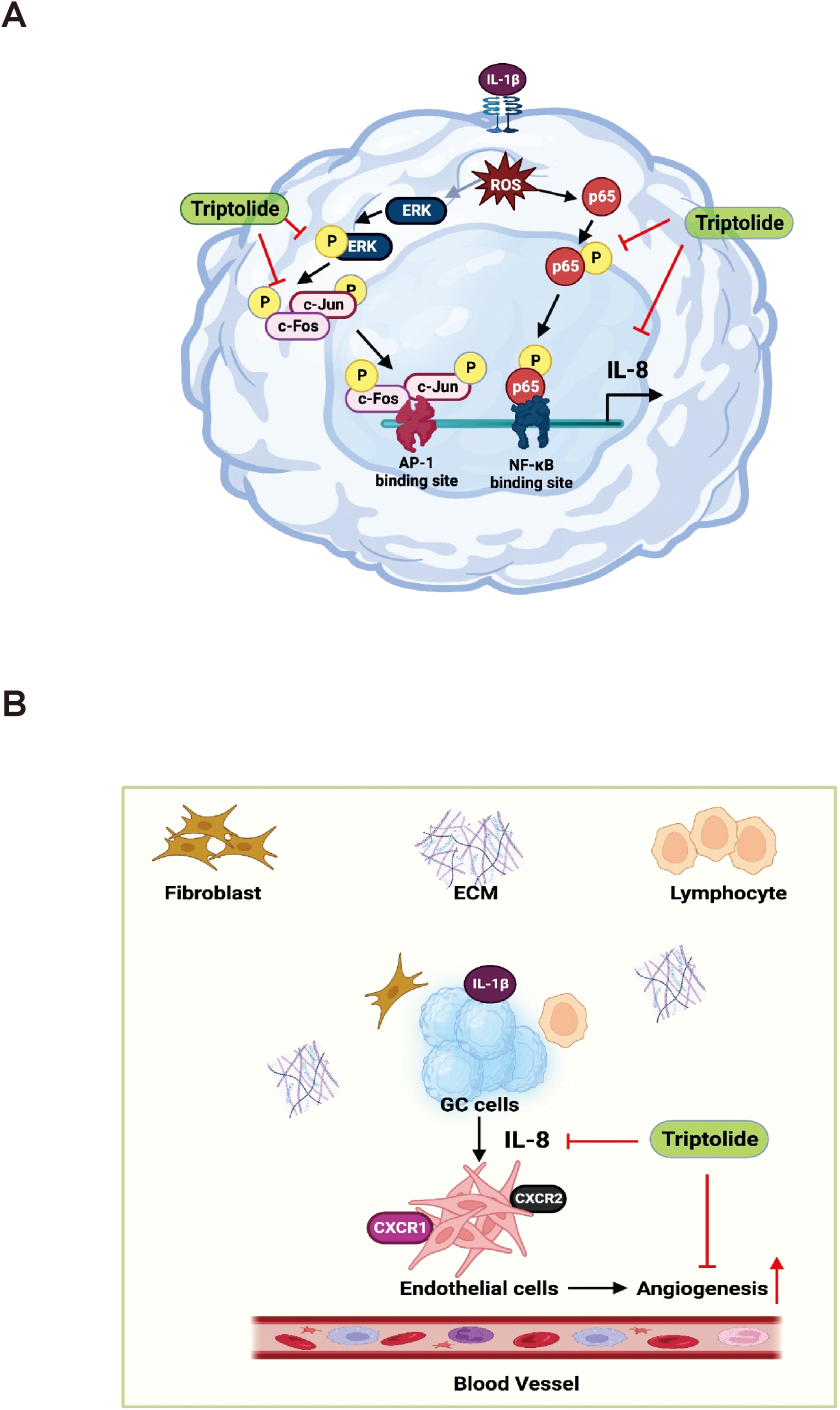
This schematic diagram illustrates the mechanisms through which Triptolide inhibits the expression of IL-8 in AGS cells, as well as the effect of AGS-derived angiogenesis on the tumor microenvironment. **(A)** Triptolide suppresses IL-8 expression in response to the activation of IL-1β by inhibiting the transcriptional activity of AP-1 and NF-KB, which mediate ROS-driven ERK1/2 signaling. **(B)** In the tumor microenvironment, AGS-derived IL-8 affects the angiogenic activity of endothelial cells.

One of the most promising novel cancer treatment strategies is targeting angiogenic pathway. *In vivo* and *in vitro* studies on GC are ongoing, and the results of these studies are highly anticipated. Angiogenesis has been identified as a cancer hallmark required for tumor survival and tumor spread to a new location (65). IL-8 produced by tumor-infiltrating macrophages is reported as a proangiogenic factor that promotes angiogenesis in various cancers (21, 66, 67). However, whether IL-8 promotes angiogenesis in gastric cancer is unknown. Our results showed that IL-1β-induced IL-8 production induced angiogenesis and Triptolide treatment inhibited this angiogenesis in the EA.hy926 cell line. Liu et al. reported that Triptolide inhibits angiogenesis via the ERK1/2-HIF1-α-VEGFA axis (68). Similarly, Xiangying Kong et al. reported that Triptolide significantly decreased the expression of angiogenic activators, including IL-17, TNF-α, VEGF, VEGFR, Tie2, Ang-1, and Ang-2. Moreover, Triptolide suppressed the IL-1β-induced phosphorylation of ERK, p38, and JNK at the protein level in the collagen-induced arthritis (CIA) DA rat model (69). To summarize, Triptolide treatment inhibited the IL-1β-induced IL-8 expression by suppressing the ERK/AP-1/NF-κB signaling, which decreased ROS generation and tumor proliferation and angiogenesis of human gastric cancer AGS cells (**Figure 6**).

Based on the data presented throughout this study, we can conclude that Triptolide has a potent chemopreventive effect and could be used as a novel therapeutic strategic approach for gastric cancer.

## 5 Conclusion

As a result of the significant health burden associated with gastric cancer, it is essential to identify effective biomarkers for predicting prognosis and determining the optimal therapeutic strategy. Triptolide, a naturally occurring compound, is critical in preventing gastric cancer development. It achieves this by inhibiting oxidative stress, downregulating the ERK, AP-1, and NF-KB signaling pathways, and inhibiting angiogenesis activity. Therefore, Triptolide’s anticancer properties and its potential as a chemopreventive agent warrant additional clinical research.

## Data Availability

The original contributions presented in the study are included in the article/**Supplementary Material**. Further inquiries can be directed to the corresponding author/s.
