# Supplementary material for: Triptolide suppresses IL-1β-induced expression of interleukin-8 by inhibiting ROS-Mediated ERK, AP-1, and NF-κB molecules in human gastric cancer AGS cells
Source: Front Oncol. 2025 Jan 30;14:1498213. doi: 10.3389/fonc.2024.1498213 (PMC11821500; doi:10.3389/fonc.2024.1498213)

Figure S1

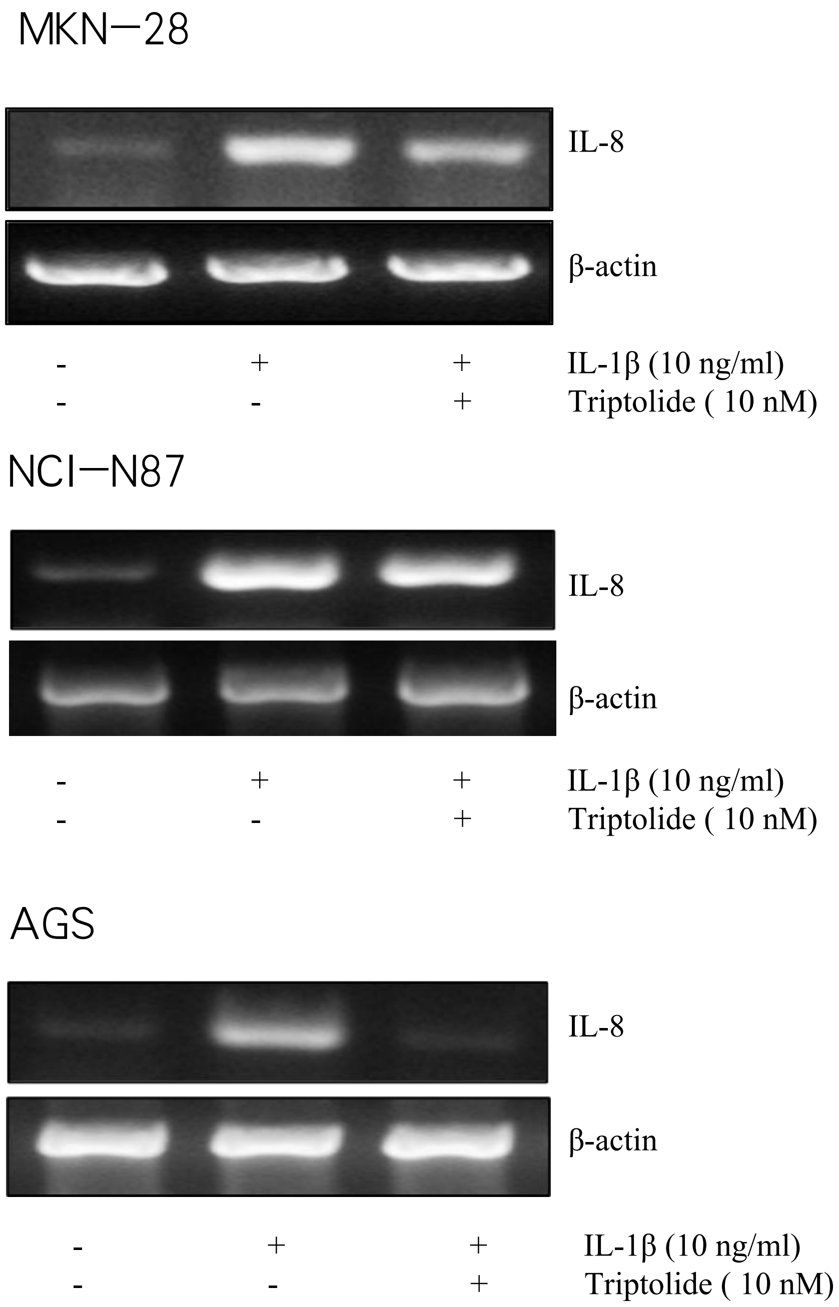

MKN−28
IL-8
β-actin
- + + IL-1β (10 ng/ml)
- - + Triptolide ( 10 nM)
NCI−N87
IL-8
β-actin
- + + IL-1β (10 ng/ml)
- - + Triptolide ( 10 nM)
AGS
IL-8
β-actin
- + + IL-1β (10 ng/ml)
- - + Triptolide ( 10 nM)

Figure S1

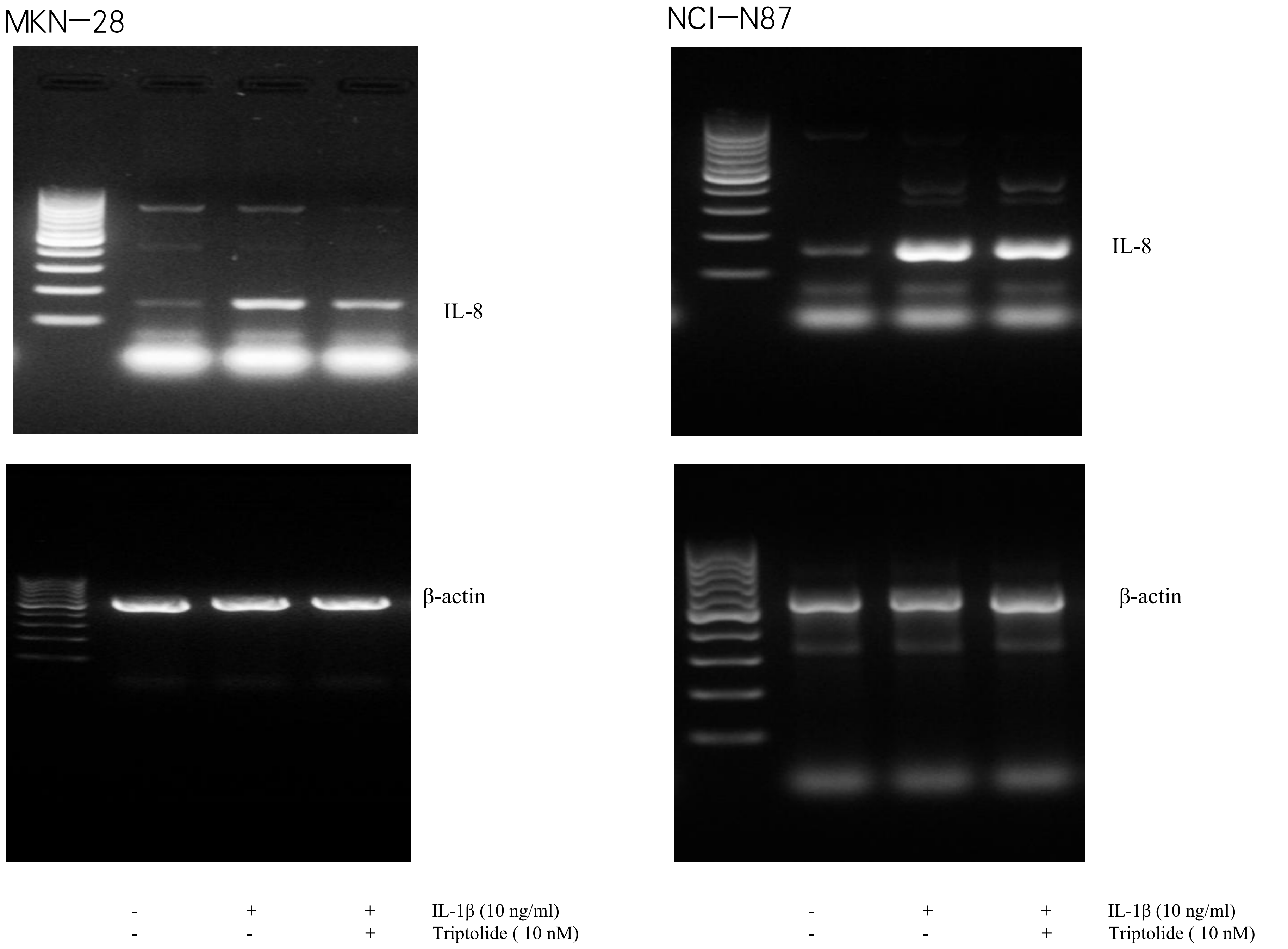
MKN−28
NCI−N87
IL-8
IL-8
β-actin
β-actin
- + + IL-1β (10 ng/ml)
- - + Triptolide ( 10 nM)
- + + IL-1β (10 ng/ml)
- - + Triptolide ( 10 nM)

Figure S1

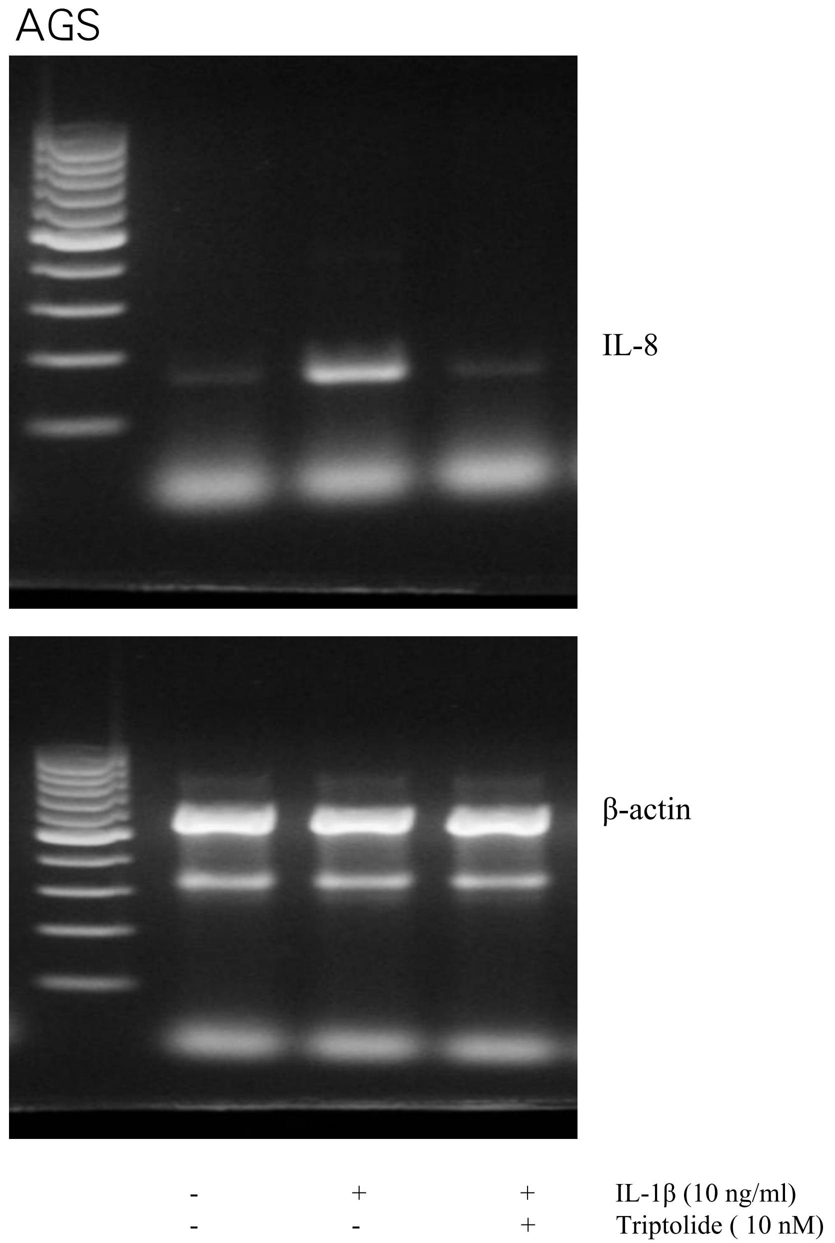

Figure S2

AGS Cell Viability

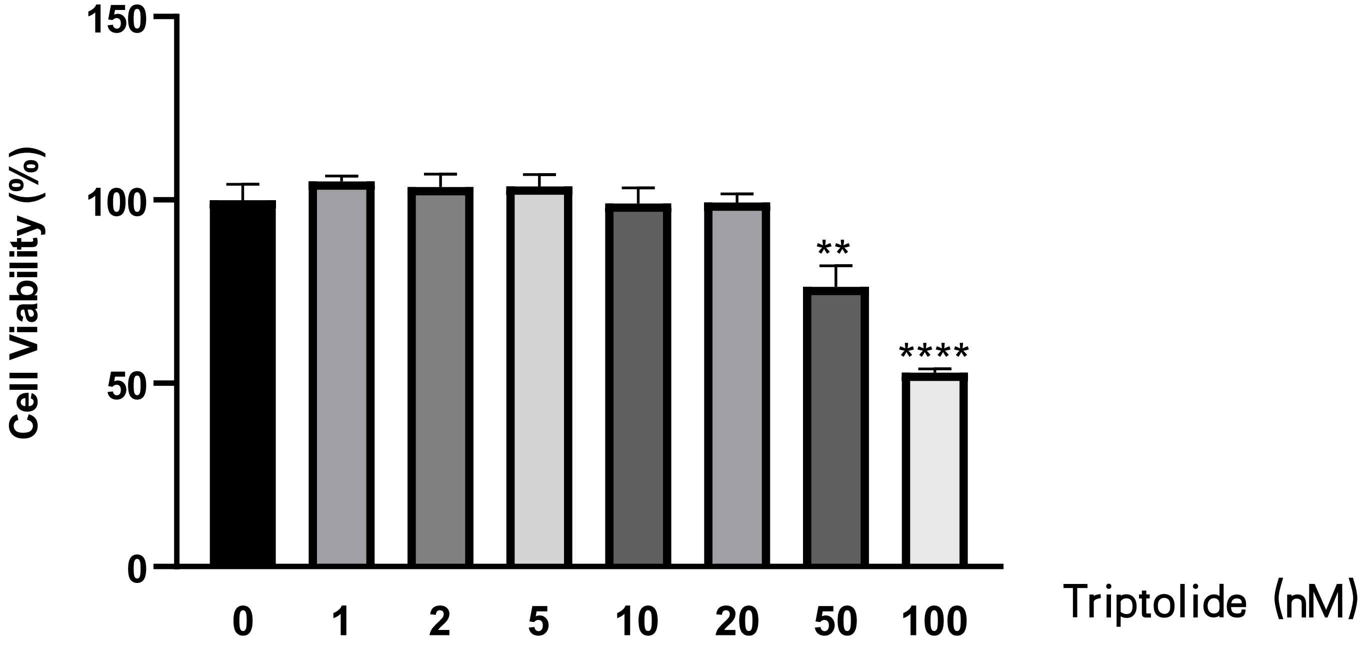

# Figure 1B

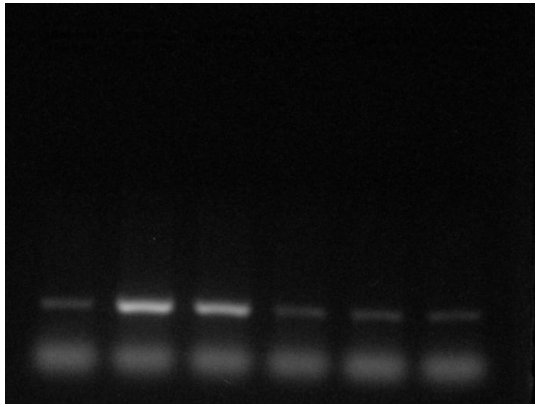

IL-8
- + + + + + IL-1β (10ng/mL)
- - 2 5 10 20 Triptolide (nM)


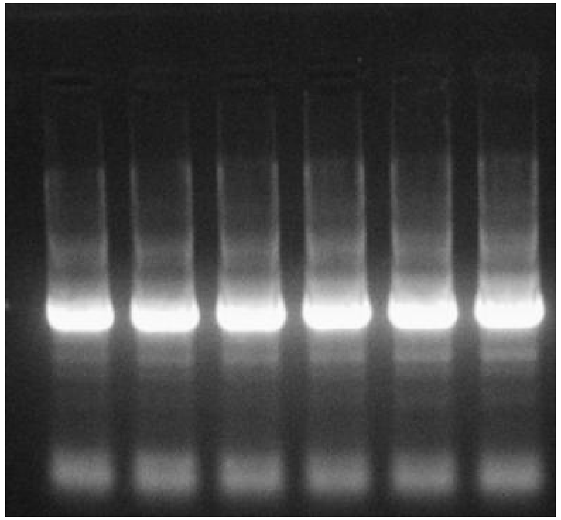

β-actin
- + + + + + IL-1β (10ng/mL)
- - 2 5 10 20 Triptolide (nM)

# Figure 2C

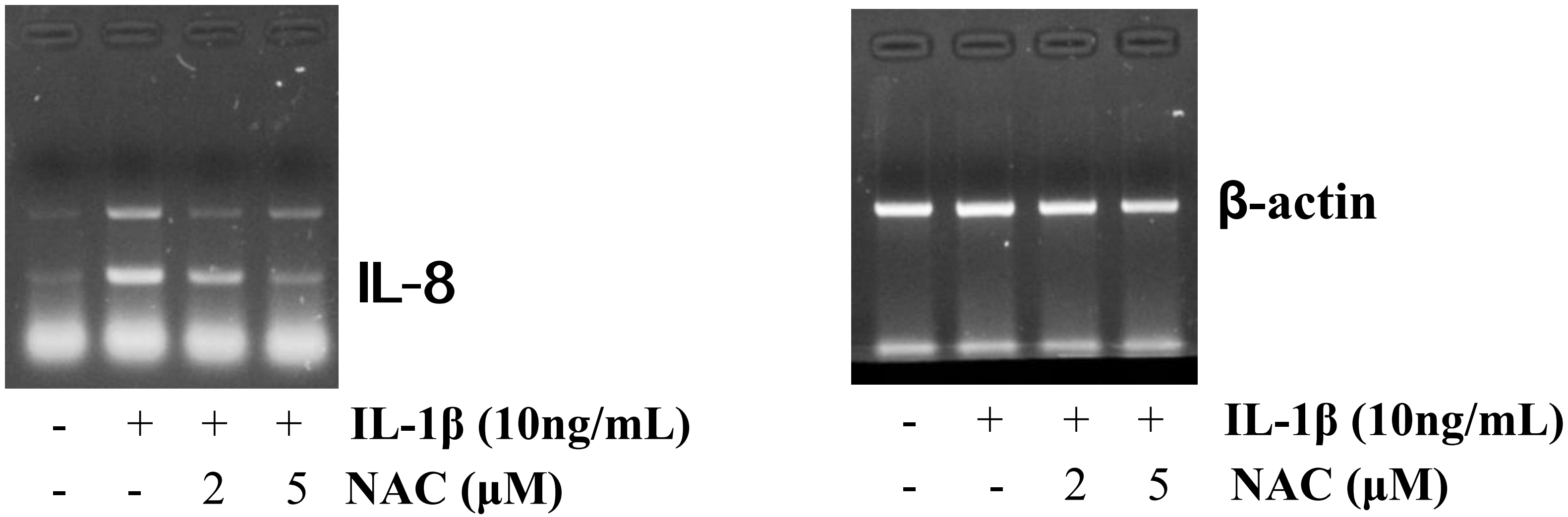

# Figure 3B

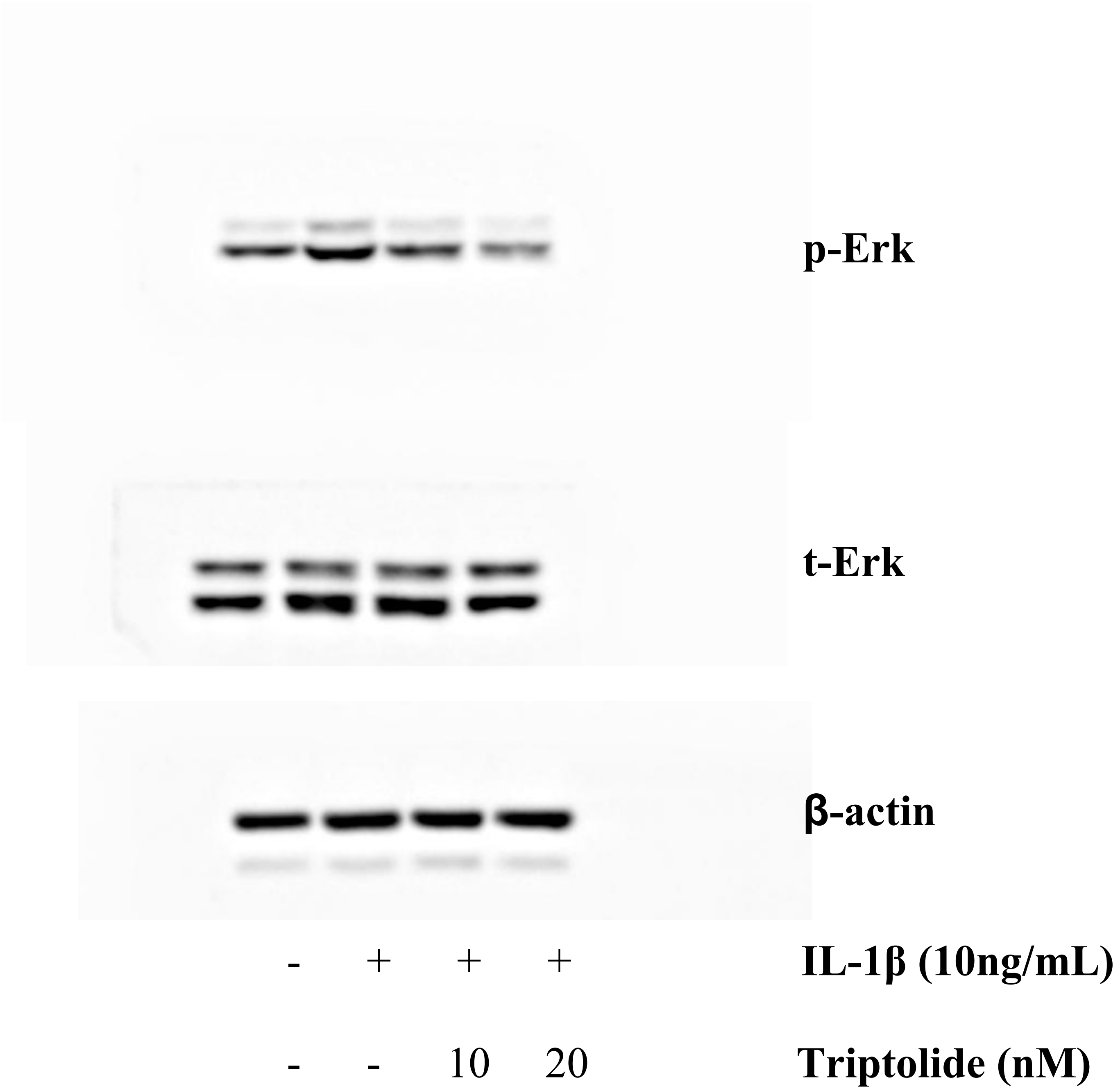

## Figure 3D

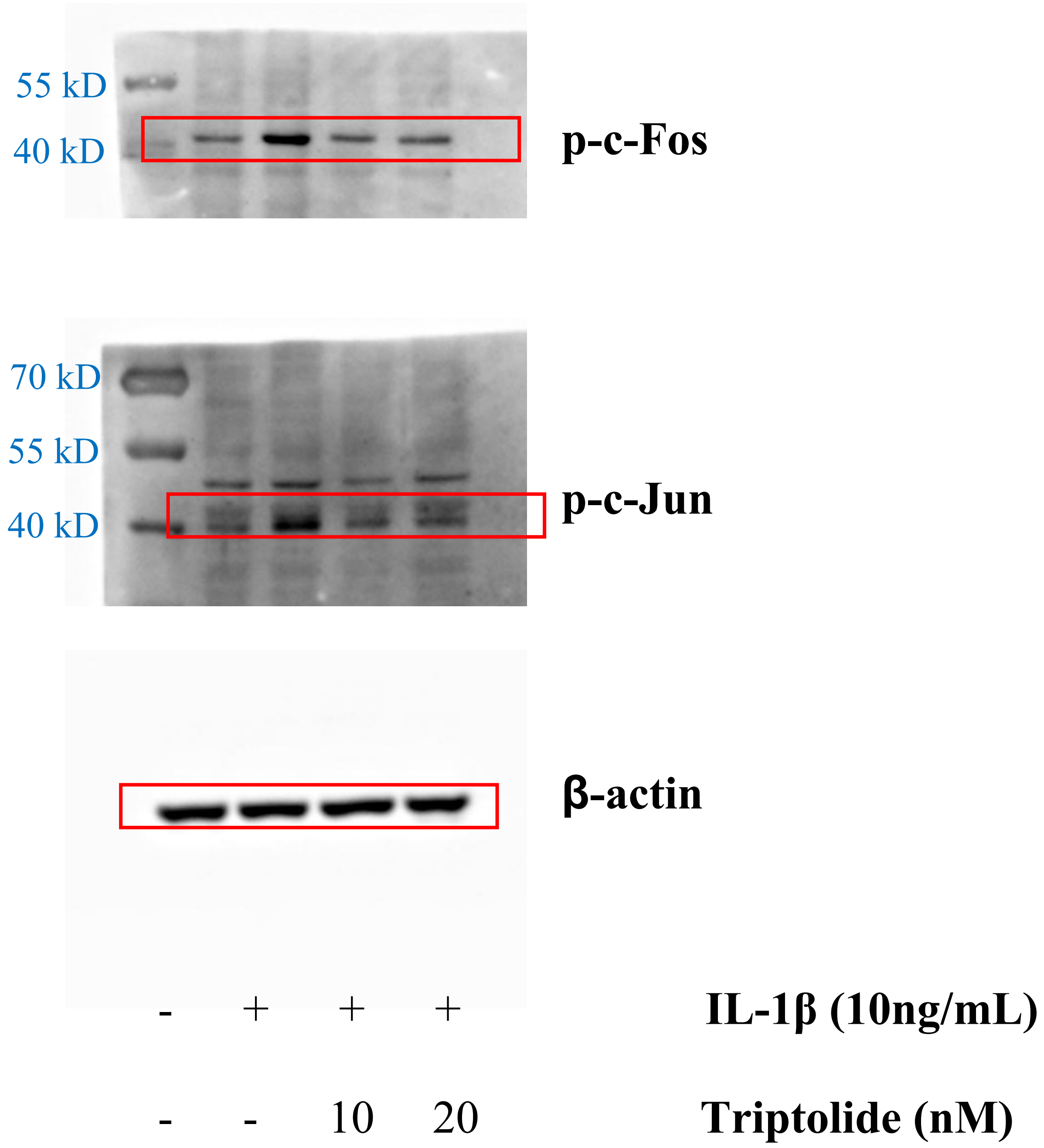

55 kD
40 kD
p-c-Fos
70 kD
55 kD
40 kD
p-c-Jun
β-actin
- + + + IL-1β (10ng/mL)
- - 10 20 Triptolide (nM)

# Figure 4D

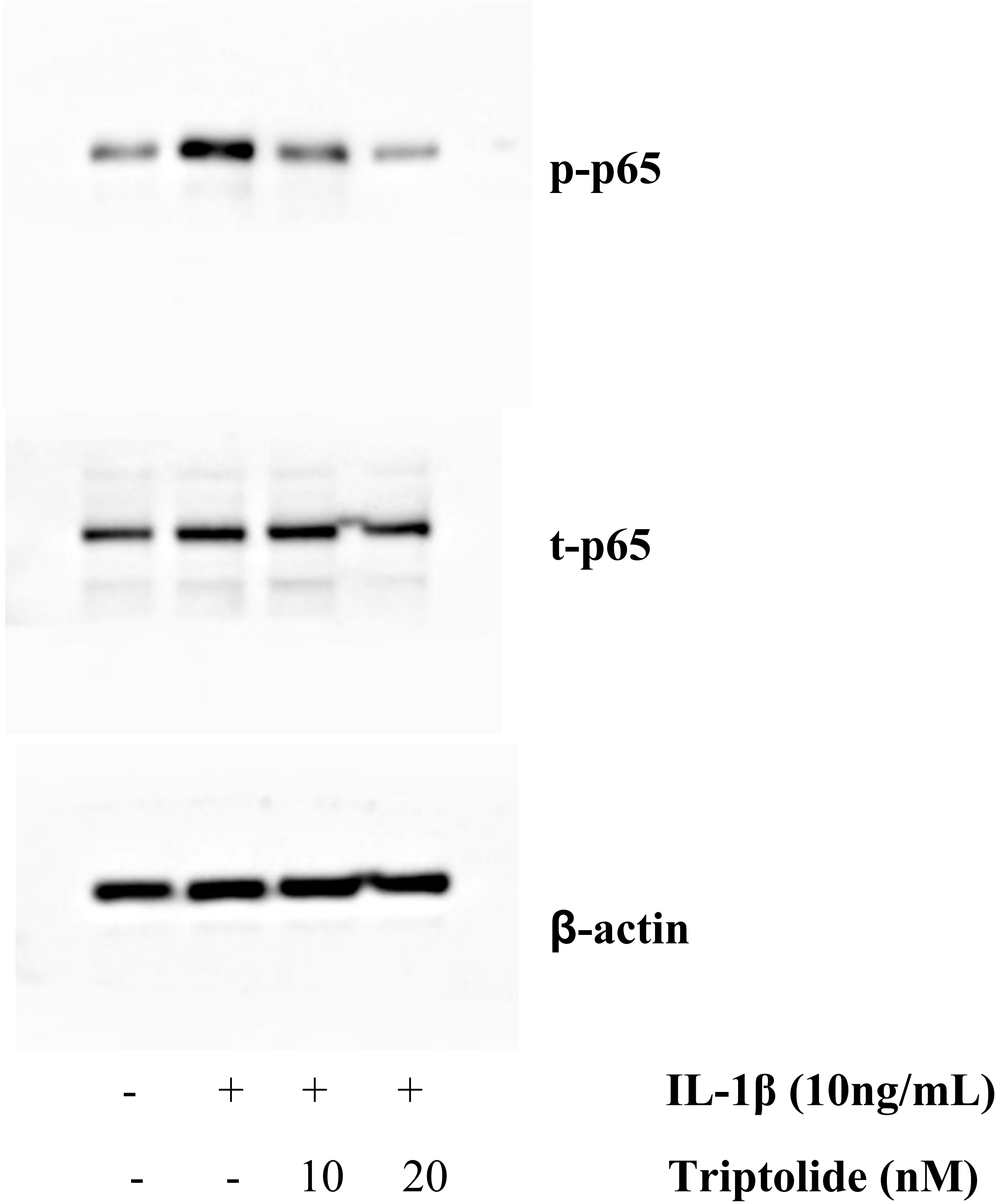

Supplement: Supplementary file 1 [file DataSheet1.pdf]
